# Aging and Renal Disease: Old Questions for New Challenges

**DOI:** 10.14336/AD.2020.0703

**Published:** 2021-04-01

**Authors:** Yu-Hsiang Chou, Yung-Ming Chen

**Affiliations:** ^1^Department of Internal Medicine, National Taiwan University Hospital Jin-Shan Branch, New Taipei City 20844, Taiwan.; ^2^Renal Division, Department of Internal Medicine, and; ^3^Department of Geriatrics and Gerontology, National Taiwan University Hospital, College of Medicine, National Taiwan University, Taiwan.

**Keywords:** aging, elderly, chronic kidney disease, acute kidney injury, end stage renal disease, dialysis

## Abstract

Chronic kidney disease (CKD) is a growing problem among aging population, and the number of individuals at risk of end stage renal disease is rising. Part of the reason lies in incomplete understanding of the pathways underlying renal aging and kidney disease, as well as insufficient delivery of evidence-based treatment to elderly patients with CKD. This review aims to address these unsolved issues by delineating updated mechanisms of renal senescence and summarizing recent findings on key clinical aspects of CKD in the elderly. Challenges and obstacles in caring for older people with CKD are discussed, with an emphasis on modification of risk factors, prevention of acute kidney injury, stabilization of progression and decision on dialysis initiation.

Chronic kidney disease (CKD) is increasingly being recognized among the elderly population, which pose significant challenges for clinicians around the world [1-3]. Many elderly people are diagnosed with CKD based merely on declining estimated glomerular filtration rate (eGFR) [4], but whether this is a process of normal aging or disease development remains controversial. In this review, we will first delineate differences between renal aging and kidney disease, and describe potential pathways and mechanisms underlying renal aging. These are followed by discussion of recent findings regarding the epidemiology, risk factors, progression and outcomes of CKD in the elderly. Finally, updated recommendations about the care of older patients with CKD are provided, with an emphasis on modification of risk factors, prevention of acute kidney injury, stabilization of renal progression and decision on dialysis initiation.

## Renal aging *versus* kidney disease

As people age, renal mass declines by about 10% per decade from aged 30 to 80 years old [5]. The number of functional nephrons also decreases, in association with a reduction of renal cortical thickness by 10% per older decade of age [6]. Renal blood flow decreases by approximately 10% per decade, along with a decline in GFR at a similar rate after age 40 [7]. Single-nephron GFR and glomerular volume, however, remain relatively constant with healthy aging [8]. Table 1 summarizes structural and functional changes of the aging kidney in the absence of overt anatomical or urinary abnormalities [9, 10].

**Table 1:** Structural and functional changes of the aging kidney.

Structural changes	Functional changes
Glomerulus • Decreased number of glomeruli • Increased glomerulosclerosis (focal and global, but not segmental) • Progressive decrease followed by increase in size of glomeruli • Shunts formation between afferent and efferent arterioles • Thickening of glomerular basement membrane • Increased mesangial volume and matrix Tubule • Decreased tubular number, volume, and length • Tubular atrophy with simplification of tubular epithelium and thickening of tubular basement membrane • Increased number of tubular diverticuli • Acquired cysts Interstitium & vasculature • Increased interstitial volume and interstitial fibrosis • Pericapsular fibrosis • Arteriosclerosis	• GFR decline • Stable single nephron GFR • Stable and minimal urinary albumin excretion • Decreased renal blood flow • Decreased sodium resorption • Decreased potassium excretion • Decreased urine concentrating capacity • Increased renal sympathetic tone • Decreased nitric oxide production • Reduced hemodynamic response to vasodilating stimulus

Studies of healthy living kidney transplant donors have provided appropriate information on both structural and functional changes that occur with normal aging. Because kidney donors undergo a sequential of clinical evaluations, including renal function and image study to confirm health before donation. Pre-implantational biopsy of the renal allograft may provide renal tissues for structural evaluation. For example, in an observational study involving 1203 adult living kidney donors, Rule et al. observed a rising prevalence of nephrosclerosis with aging, from 2.7% for ages 18-29 years to 73% for ages 70-77 years, as determined by core needle biopsy of the graft kidney. They found a strong association between age and nephrosclerosis even after adjustment for kidney function and risk factor covariates such as diabetes and hypertension [11].

The diagnosis of CKD by eGFR <60 mL/min in older people has been criticizing for overestimating the CKD burden in the elderly population because eGFR generally declines inversely with aging, and reduction of eGFR to 50-59 mL/min/1.73 m^2^ does not increase mortality risk among patients ≧65 years compared to patients with eGFR of more than 60 mL/min/1.73 m^2^ [12, 13]. Alternatively, a meta-analysis performed by Coresh et al. argued that even smaller decreases in eGFR could be associated with increased mortality and risk of end-stage renal disease (ESRD) [14]. This raises uncertainty about using the traditional eGFR thresholds of CKD to predict outcomes for the elderly. Therefore, there is a need for a more sensitive indicator or formula to estimate kidney function in the elderly [15]. Recently, Delanaye et al. called for age-adapted definition of CKD to avoid inappropriate care. They emphasized when using eGFR definition for CKD diagnosis, CKD should be defined with respect to clinical outcomes or complications at the age-specific thresholds for eGFR. This argumentation is supported by observations that GFR may decline with aging without any sign of kidney damage for older living kidney donors, and the risk of mortality increases at higher eGFR among younger people than in elderly individuals [16, 17].

Despite controversy over definition, the prevalence of CKD increases dramatically from about 5% in younger adults (aged 20-39 years) to nearly half of older adults (aged 70 years and above) [18]. This age-gradient can be partially explained by age-related decline in GFR, without major metabolic, vascular or immunologic derangements. Indeed, it has been demonstrated that earlier stages of CKD in the elderly are more likely due to age-associated decline in glomerular filtration than real kidney disease [19]. Some studies even suggest older people with CKD may exhibit slower progression and better outcomes [20-23]. For example, in the United States, among patients with eGFR levels <45 mL/min per 1.73 m^2^, older patients were less likely to have an annual decline in eGFR of >3 mL/min per 1.73 m^2^ compared to younger patients [20]. In Europe, a collaborative study enrolling 1248 patients with stage 3-4 CKD, the risk of ESRD was higher than the risk of death without ESRD for ages <60 years, independent of eGFR levels. The ESRD risk diminished with aging except in patients between 65 and 75 years with eGFRs of 25-35 mL/min per 1.73 m^2^, and in patients up to 85 years with an eGFR <15 mL/min per 1.73 m^2^ [21]. In the United Kingdom, a prospective observational study comparing the impact of old age on outcomes in 2,667 CKD patients with eGFR <60 mL/min/1.73 m^2^ found those under 55 years had higher risk of renal replacement therapy (RRT) than death, compared to those >75 years [22]. Consistent with the above results, we have observed that among 430 patients with stage 3-5 CKD, those aged between 20-39 years and 40-64 years, compared to ≥75 years, exhibited a higher risk of ESRD. This finding suggests old age could be a positive modifier of renal outcome, independently of eGFR and proteinuria [23]. That being said, there is no denying that the incidence of ESRD has been highest among older people, in particular those aged 75 years or over [24, 25]. As populations are getting older at a faster pace, further studies are needed to elucidate this paradox and determine whether differences in case mix or co-morbid burdens are involved in variability of ESRD risk associated with older age.

### Specific mechanisms for renal aging

### Renin-angiotensin system (RAS) activity

In humans, studies have shown a progressive decline in systemic RAS activity with aging, as represented by plasma renin and aldosterone levels [26-28]. Conversely, in experimental animals, the activity of intrarenal RAS, as reflected by urinary angiotensinogen [29], increased despite a decline in the circulating RAS. Additionally, intravenous infusion of angiotensin I led to reduction in glomerular filtration and plasma flow rates, especially in older animals [30]. Such increased sensitivity to RAS stimulation may exacerbate age-associated decline of renal function in the face of RAS stimuli such as hypovolemia, hypotension or sodium restriction. Changes in intrarenal RAS activity with aging may also precipitate an altered response to RAS blockade. For example, the protective effects of angiotensin-converting enzyme (ACE) inhibitors on glomerulosclerosis and albuminuria are blunted in aged animals [31]. And the hypotensive effect exerted by these drugs are less prominent in older hypertensive patients compared to younger counterparts [32].

Apart from its effect on hemodynamics, age-related RAS dysregulation has been implicated in mitochondrial reduction-oxidation (redox) changes and the formation of excessive free radicals, both of which play a part in renal fibrogenesis [33]. In the kidney, knockout of the *Agtr1a* gene that encodes angiotensin II type 1a (AT1a) receptor is associated with an increased number of mitochondria and upregulation of the prosurvival genes nicotinamide phosphoribosyltransferase (*Nampt*) and sirtuin 3 (*Sirt3*) [34]. And RAS blockade with enalapril and losartan protected against mitochondrial dysfunction in aged animals [35]. The mitochondrial-antioxidant contents including nitric oxide synthase, manganese-superoxide dismutase and cytochrome oxidase activities, and uncoupling protein-2 are higher in spontaneously hypertensive rats treated with angiotensin II receptor blockers (ARBs) than those with Ca^2+^ channel blockade [36]. Moreover, another prosurvival factor, sirtuin 1 (SIRT1), has been shown to attenuate oxidative stress in aged kidneys through suppression of AT1 receptor expression both *in vitro* and *in vivo* [37]. Mechanistic studies in human glomerular mesangial cells have also demonstrated that angiotensin II induces the shortening of telomere lengths, P53 and P21 expression, as well as cell cycle arrest. All of these effects are major mechanisms of cell senescence and could be overcome by the treatment with ARB [38].

### Chronic inflammation

Numerous studies have demonstrated that age-related dysregulation of the immune system, so called immunosenescence, is prevalent in normal older individuals. For instance, healthy elderly adults have increased levels of interleukin (IL)-1β, IL-6, IL- 8, IL-18, C-reactive protein, and tumor necrosis factor-α, as well as a decrease in IL-10 [39, 40]. The elderly also shows a shift response from a T helper (Th)-1 cytokine to a Th-2 cytokine [41], accompanied by an increase in Th-17 cells and a decrease in regulatory T cells [42]. Additionally, several inflammasomes have been identified in the aging kidneys, in association with high levels of toll-like receptor-4 and IL-1 receptor. The expression of inflammasome components such as nucleotide-binding domain leucine-rich repeat (NLR), NLR pyrin domain-containing protein 3 (NLRP3), NLR family caspase recruitment domain-containing protein 4 (NLRC4), and pro-caspase-1 are significantly upregulated in the aging rat kidney [43]. Another study by Sato et al. showed that superaged mice would spontaneously develop renal tertiary lymphoid tissues (TLTs), which may promote inflammatory and fibrotic responses in the kidney [44]. All of these changes causing imbalance of inflammatory cells and associated cytokines are known to aggravate age-related decline in renal function [45, 46].

### Dysfunctional signal transduction

#### Klotho and Wnt/β-catenin signaling

The anti-aging protein, Klotho, is highly expressed in the tubular epithelium of normal adult kidneys and plays a crucial role in modulating diverse age-associated pathways. For example, Klotho is an antagonist of Wnt/β-catenin signaling whose activation promotes renal fibrogenesis. Overexpression of Klotho can also abolish the fibrogenic effects of transforming growth factor-β1 [47]. As Klotho levels declines with aging, Wnt/β-catenin signaling increases, promoting renal fibrosis and vascular calcification [48-50].

#### Peroxisome proliferator-activated receptor (PPAR)-γ

Peroxisome proliferator-activated receptor-γ (PPAR-γ) is a nuclear receptor with activity that decreases with aging [51]. PPAR-γ agonists not only increase Klotho expression but also reduce oxidative stress and improve vascular function in aging rats [52]. Increased PPAR-γ expression by pioglitazone or baicalin suppresses age-associated inflammation through blocking pro-inflammatory nuclear factor-κB (NF-κB) activation in aged rat kidney [53].

### Oxidative stress

Aging is associated with increased oxidative stress which in turn results in tissue damage and accelerates aging process [54]. Increased mitochondrial oxidative phosphorylation, as well as reduced levels of catalase, Cu/Zn-superoxide dismutase, and glutathione reductase have been linked with a reduced antioxidant capacity in the aging kidney [55, 56]. All of these changes impair the electron transport chain in mitochondria, leading to the progressive failure of mitochondrial bioenergetics and homeostasis [57]. SIRT1 is a nicotinamide adenine dinucleotide^+^-dependent deacetylase which plays a role in redox defense, but its level is reduced with aging. Kume et al. demonstrated that caloric restriction protects the aging kidney by preserving renal *Sirt1* expression and preventing mitochondrial autophagy [58]. Augmented expression of *Sirt1* could enhance the ability of renal interstitial cells to resist the oxidizing medullary environment and produce antifibrotic and antiapoptotic actions [59]. Another study by Chuang et al. confirmed that podocyte-specific *Sirt1* knockdown mice were associated with reduced activation of the PPAR-γ coactivator-1α (PGC-1α), forkhead box O3, forkhead box O4, and p65 NF-κB, through SIRT1-mediated deacetylation. These suggest that dysregulation of SIRT1 results in activation of downstream pathways of oxidant stress, cell death, and inflammation in podocytes [60]. SIRT3 is another mitochondrial deacetylase that can increase mitochondria number and upregulate the prosurvival genes, thereby protecting cells from oxidative stress-mediated injury [61]. Benigni et al. found that angiotensin II was able to downregulate *Sirt3* mRNA expression in cultured tubular epithelial cells and this effect could be inhibited by AT1 receptor antagonist [34]. Together, the sirtuin family of proteins may be a potential target for patients with age-related decline in kidney function.

### Vascular changes

Aging kidneys are characterized by progressive narrowing of renal vasculature as exemplified by the prevalence of renal artery narrowing or atherosclerosis which increases from 0.4% in the 18-29 years age group to 25% in the 60-76 years age group [62]. At the microvascular level, increased shunting of cortical blood to the medullary circulation, atrophy of afferent and efferent vessels, and loss of peritubular capillaries lead to a gradual reduction in the circulation of nephron [63]. Using "hypoxia-responsive" reporter of the transgenic rats, age-related expansion of hypoxia was identified in all areas of the kidney, particularly in the cortex. The degree of hypoxia correlated with upregulation of vascular endothelial growth factor and glucose transporter-1, highlighting the involvement of hypoxia-inducible factor and its target genes in the aging kidney [64].

There is a tendency toward developing disproportionate vasoconstriction of the renal vasculature in the elderly. This is likely caused by increased sympathetic tone and propensity to vasoconstrictor effects of angiotensin II, endothelin, and platelet-activating factor, as well as decreased response to renal vasodilators, such as atrial natriuretic peptide, nitric oxide, and amino acids. Such imbalance which is in favor of vasoconstrictive force makes the old kidney difficult to maintain its normal renal plasma flow, thereby increasing susceptibility to nephrotoxic injury [65, 66].

### Telomere shortening

Although telomere length declines with age, it remains controversial whether telomere length reflects the byproduct of aging or a biomarker that can influence biological conditions, delay senescence and promote longevity [67]. Most studies favor that telomere shortening is the result of aging which indicate the state of cell senescence. However, some studies point out that telomere shortening makes the aged kidney more vulnerable to injury and causing maladaptive repair. Telomerase is a reverse transcriptase enzyme complex that adds DNA sequence repeats to DNA strands in the telomere regions. Telomerase contains 2 major components, TerT and TerC, which together prevent telomere shortening. Telomerase gene therapy has been shown to delay aging and increase longevity in aged mice [68]. Some studies have also demonstrated delayed recovery from renal ischemia-reperfusion injury in mice with deletion of either TerC or TerT, suggesting a crucial role of telomerase on renal regeneration after injury [69].

## Epidemiology, risk factors, progression and outcomes of CKD in older patients

Several large-scale studies have reported the prevalence of CKD increases disproportionately with age [18, 70]. The National Health and Nutrition Examination Survey (NHANES) during 1999 and 2004 in the United States reported 46.8% of people aged 70 and older, compared to 6.71% in those between 40-59 years of age, had moderately severe CKD as defined by an eGFR <60 mL/min/1.73 m^2^calculated by the Chronic Kidney Disease Epidemiology Collaboration (CKD-EPI) equation [18]. The National Kidney Foundation's Kidney Early Evaluation Program (KEEP) during 2000 and 2008 also reported the prevalence of CKD in people 65 years and older was approximately 44%, with the highest representation being observed in those over age 80. Most of them (77%) belonged to CKD stage 3 and only 5% were stage 4 [70]. In other countries, the prevalence of CKD in the elderly population is similarly high, with as many as 30.5% of Beijing citizens older than 70 years, and 30.8% of Canadian older than 65 years showing eGFR <60 mL/min/1.73 m^2^ or markers of kidney damage [71, 72]. In a systematic review comprising 26 population-based studies, the researchers concluded that the prevalence of CKD in people aged 64 years or older was between 23.4% and 35.8% [73]. Consistent with these observations, a prospective cohort study based on 462,293 adults who participated in a standard medical screening program during 1994 and 2006 in Taiwan found 37.2% of participants aged 65 years or over had an eGFR <60 mL/min/1.73 m^2^ [74]. Another prospective cohort study integrating community-based screening program of 106,094 adults in northern Taiwan confirmed the high prevalence of total CKD (15.46%) and CKD stages 3-5 (9.06%), which occurs predominantly in older individuals [75].

**Table 2:** Risk factors for chronic kidney disease in the elderly.

Modifiable risk factors	Non-modifiable risk factors
• Acute kidney injury (perioperative cardiac dysfunction, sepsis, obstructive uropathy, hypovolemia, NSAID use, radiocontrast media, pauci-immune glomerulonephritis) • Cardiovascular disease (heart failure, renal artery stenosis) • Diabetes mellitus • Hypertension • Proteinuria • Obesity • Hyperlipidemia • Hyperuricemia • Smoking	• Demographics (age, gender) • Race & ethnicity • Low birth weight • Family aggregation • Hereditary disease

Only a few studies have focused on risk factors for development of CKD in older adults, despite a plethora of data within general population (Table 2) [76, 77]. As mentioned earlier, CKD is a common disorder among aging populations and the cause of which is likely multifactorial. The presence of diabetes, hypertension and glomerulonephritis, dyslipidemia and cardiovascular diseases, as well as inappropriate use of non-steroidal anti-inflammatory drugs (NSAIDs) or herbal medicine all play a part in triggering damage to the aged kidney with decreased physiological reserve [78]. Additionally, acute kidney injury (AKI), which often occurs during the course of acute illnesses is becoming a prominent risk factor for subsequent development of CKD and ESRD [79, 80]. More and more patients with critical conditions are saved at the expense of AKI. In a systematic review and meta-analysis comprising 17 studies of patients with AKI, 31.3% of surviving elderly patients (≧65 years) did not recover kidney function compared to 26% of younger patients [81]. These findings indicate elderly patients are less likely to recover from acute kidney injury (AKI), which likely reflects decreased reparative and regenerative potential of the aged kidney [77, 79, 80].

**Table 3:** Treatment goals for glycemia, blood pressure, and dyslipidemia in older adults with or without diabetes.

Diabetic patient characteristics/health status (ADA guideline, 2019)	Rationale	HbA1C goal	Fasting or preprandial glucose (mg/dL)	Bedtime glucose (mg/dL)	Blood pressure (mmHg)	Lipids
Healthy (few coexisting chronic illnesses, intact cognitive and functional status)	Longer remaining life expectancy	<7.5%	90-130	90-150	<140/80	Statin unless contraindicated or not tolerated
Complex/intermediate (multiple coexisting chronic illnesses or 2+ instrumental ADL impairments or mild to moderate cognitive impairment)	•Intermediate remaining life expectancy, •high treatment burden, •hypoglycemia vulnerability, •fall risk	<8.0%	90-150	100-180	<140/80	Statin unless contraindicated or not tolerated
Very complex/poor health (long-term care or end-stage chronic illnesses or moderate to severe cognitive impairment or 2+ ADL dependencies)	Limited remaining life expectancy makes benefit uncertain	<8.5%	100-180	110-200	<150/90	Consider likelihood of benefit with statin (secondary prevention more than primary)
Non-diabetic patient characteristics	Blood pressure (mmHg)				Reference	
< 65 y	< 130/80				ACC/AHA guideline, 2017	
≥ 65 y	< 130/80					
< 65 y	< 130/80				ESC/ESH guideline, 2018	
≥ 65 y	< 140/80					
Non-diabetic patient characteristics	LDL-C mg/dL				Reference	
40-75 y	≥70 - <190				ACC/AHA guideline, 2019	
> 75 y	Clinical assessment, risk discussion					
> 65 y	1. Treatment with statins is recommended for older people with ASCVD in the same way as for younger patients				ESC/ESH guideline, 2019	
	2. Treatment with statins is recommended for primary prevention, according to the level of risk, in older people aged ≦75 years.					
	3. Initiation of statin treatment for primary prevention in older people aged >75 years may be considered, if at high-risk or above.					
	4. The statin should be started at a low dose if there is significant renal impairment and/or the potential for drug interactions, and then titrated upwards to achieve LDL-C treatment goals.					

ADA, American Diabetes Association; ADL, Activities of daily living; ACC/AHA, American College of Cardiology/American Heart Association; ASCVD, Atherosclerotic cardiovascular disease; ESC/ESH, European Society of Cardiology/European Society of Hypertension; HbA1c, hemoglobin A1c; LDL-C, Low density lipoprotein-cholesterol.

Several studies have reported predictors of renal progression to ESRD in the elderly. Nicola et al. identified high proteinuria level as a strong prognostic factor of ESRD development in older patients with incipient CKD [21]. Obi et al. found age as a determinant of mortality, whereas overt proteinuria was strongly associated with ESRD in the elderly [82]. They further showed the incidence of ESRD was appreciably higher than that of death before RRT in patients with advanced CKD and overt proteinuria. The mechanism whereby proteinuria causes progressive kidney damage has been described elsewhere [83]. Given its clinical significance, proteinuria has been incorporated into Tangri et al.’s predictive model for progression of CKD to ESRD, the 4-variable Kidney Failure Risk Equation (KFRE) [84-86]. Based on the formula, older patients with CKD display a lower probability of renal failure, compared to younger counterparts. These findings contradict the assumption that GFR declines by 10% per decade after the fourth decade, suggesting that decline in GFR with age might not be inevitable, at least for those without concomitant diabetes or hypertension. In another study, older patients were found less likely to develop ESRD than their younger counterparts with similar levels of eGFR, even after adjustment for the competing risk of mortality [23]. Nevertheless, older people in particular those ≧75 years of age constitute the largest percentage of incident patients with ESRD. The explanation for this paradox remains unclear, although in a large representative sample from the Unites States Medicare beneficiaries, up to 25.2% of ESRD patients were identify having a prior history of AKI [87]. Therefore, from a preventive perspective, it seems imperative that every efforts should be made to provide quality care for elderly people with or without existing CKD, not only to save lives [88-91], but to maintain quality of life by abolishing the transition of AKI to CKD and ESRD.

## Treatment and care management for the elderly

Taiwan will become a super-aged society by 2026 when at least 20 percent of the population are 65 years or older (National Development Council. Population projections for Taiwan, assessed at https://pop-proj.ndc.gov.tw/main_en/ June 12, 2020). The prevalence of elderly CKD is expected to increase further if the current preventive measures against CKD remains unchanged. It has been suggested that age-specific care considerations should be incorporated into the scope of management for elderly patients with CKD [92, 93]. To name just a few, GFR thresholds for diagnosing CKD may be adjusted downward to 45-50 mL/min/1.73 m^2^, based on mortality outcomes stratified by levels of GFR in the general population [9, 94]. The prescription of NSAIDs may be subjected to tighter regulations in the elderly with CKD [95].

There are few evidence-based guidelines available for preventing CKD progression in the elderly. More studies in this regard are warranted. The following are some selected topics which merit special attention for the medical professionals.

### Modification of risk factors

For older people with or without CKD, modifiable risk factors or controllable comorbidities should be identified and managed appropriately, irrespective of diabetic status (Table 2). The American Diabetes Association has established a framework of management of glucose, blood pressure and lipids for the diabetic elderly [96, 97]. Three major classes of older patients are included: 1) relatively healthy, 2) those with complex medical histories where self-care may be difficult, and 3) those with very significant comorbid illnesses and functional impairment. As shown in Table 3, screening for diabetes and prediabetes is necessary for people over 65 years, and even earlier in overweight people over 45 years by using either a fasting plasma glucose test, hemoglobin A1c, or oral glucose tolerance test every 1-3 years. Because the elderly has vulnerability to hypoglycemia, glycemic goals for older adults may be relaxed using individualized criteria according to comorbidities they have. Treatment of hypertension and dyslipidemia should also be tailored in older adults according to the time frame of benefit, taking into account patient characteristics such as individual’s cognitive and functional status, risk for cardiovascular events and tolerance to antihypertensive drug therapy. Lipid-lowering therapy should also be used for management of dyslipidemia unless contraindicated or not tolerated. For non-diabetic elderly patients, treatment of hypertension and dyslipidemia can follow the same guidance as for diabetic counterparts [98-102]. The general principles of ‘start low and go slow’ and ‘avoid potentially inappropriate medications’ endorsed by the geriatric societies should be adopted, regardless of diabetic status, to prevent adverse drug reactions or events [98-101, 103, 104].

### Prevention of AKI

AKI, even if followed by renal recovery, is emerging as a prominent risk factor for the future development of CKD and ESRD [79, 80]. A cohort of 233,803 hospitalized, older patients without previous ESRD or AKI, showed that the hazard ratio for developing ESRD was 41.2 for patients with AKI and CKD compared to those without kidney disease. Among patients who progressed to ESRD, 25.2% had a previous history of AKI, indicating that episodes of AKI during hospitalization may accelerate progression of renal disease, particularly those with CKD [87]. Because of few proven pharmacological treatments for AKI in the elderly [105], implementation of preventive measures to protect kidney function is clearly a better strategy. Recognizing the increased vulnerability and potential risk factors of the elderly is crucial to establish pragmatic approaches to prevent AKI. Some of these include investigating applicable formula or biomarkers to detect early AKI, treating diabetes and hypertension, managing dyslipidemia and cardiovascular diseases, preventing sepsis, and avoiding contrast nephrotoxicity and abuse of NSAIDs [106]. Of note, blood pressure levels should be monitored periodically to avoid unstable hemodynamics and inadvertent AKI [107, 108], especially for those with poor medical conditions. Other general measures for the prevention of AKI are listed in Table 4.

**Table 4:** Preventive strategies for acute kidney injury in the elderly.

• Identify high risk clinical settings Preexisting CKD, cardiac failure, malnutrition, hypovolemia, perioperative period of cardiovascular surgery
• Avoid nephrotoxic insults Prevent contrast-induced nephropathy Restrain frequent radiocontrast-based procedures Use lowest dose in the shortest time possible (analgesics)
• Keep optimal hemodynamics during treatment of hypertension Avoid use of diuretics and/or NSAIDs on top of ACE inhibitors or ARBs Target systolic blood pressure level around 120-140 mmHg
• Prevent sepsis development by timely and effective antimicrobial therapy

### Stabilization of CKD progression

Novel drug development for kidney diseases has been limited over the last few decades. Current practice guidelines recommend blood pressure control with RAS blockade as the gold standard therapy for patients with proteinuric CKD. However, blocking the RAS by either ACE inhibitors or ARBs slows but does not stop the progression of kidney disease [80, 109-111]. The old drug pentoxifylline, a nonselective phosphodiesterase inhibitor, could reduce the occurrence of AKI as determined by attenuation of serum creatinine rise [112, 113], associate with reduction of neonatal sepsis related metabolic acidosis [114], and display add-on renoprotective activities when administered in conjunction with RAS blockade [110, 111]. The current anti-diabetic agent, PPAR-γ agonist may have some protective effects on kidney diseases and renal aging as well as amelioration of hyperuricemic nephropathy [115-118]. Sodium-glucose cotransporter-2 (SGLT2) inhibitors are a new class of glucose-lowering medications that have been approved for use in patients with type 2 diabetes. These drugs have been shown to exert beneficial effects on cardiovascular events and renal endpoints, possibly through extra-glycemic mechanisms [119]. SGLT2 inhibition increases glucose and sodium delivery to the macula densa, which induces the tubuloglomerular feedback. This effect results in afferent arteriole vasoconstriction and a reduction in intraglomerular pressure [120]. Another multinational observational cohort study reported that using SGLT2 inhibitor therapy was associated with a lower eGFR decline and lower risk of major kidney events compared with initiation of other glucose-lowering drugs [121]. Importantly, trials of SGLT2 inhibitors in non-diabetic progressive kidney disease are in progress [122]. (DAPA-CKD, A Study to Evaluate the Effect of Dapagliflozin on Renal Outcomes and Cardiovascular Mortality in Patients with Chronic Kidney Disease; EMPA-KIDNEY, The Study of Heart and Kidney Protection with Empagliflozin). No subgroup analyses have yet been performed in older people to explore the effects of SGLT2 inhibitors to prevent CKD development or progression, irrespective of diabetic status. More novel therapeutic agents such as Klotho agonists and drugs targeting cellular senescence including navitoclax, dasatinib and quercetin have been developed but more investigations are needed to confirm their clinical utility [123, 124].

### Decision on dialysis initiation

**Figure 1.**
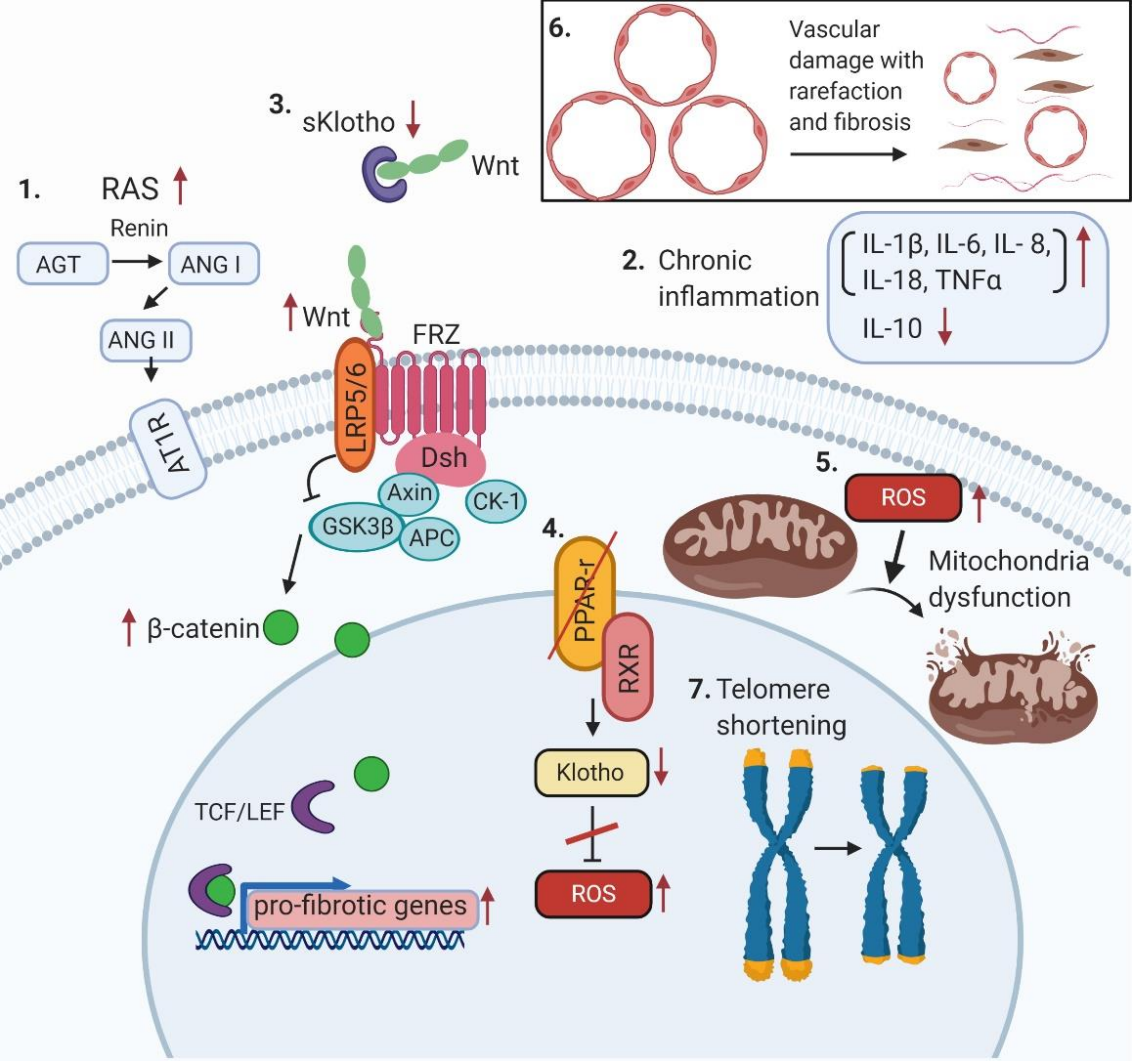
Molecular mechanisms of renal aging. (1) Activation of RAS system (2) Chronic inflammation (3) Decreased Klotho expression and activation of Wnt/β-catenin signaling (4) Decreased PPAR-γ activity (5) Increased oxidative stress and mitochondrial dysfunction (6) Vascular damage (7) Telomere shortening. Abbreviations: AGT, angiotensinogen; ANG I, angiotensin I; ANG II, angiotensin II; AT1R, angiotensin II type 1 receptor; FRZ, Frizzled family receptor; GSK, glycogen synthase kinase; LRP5/6, lipoprotein receptor-related protein 5/6; RXR, retinoid X receptor; ROS, reactive oxygen species; TCF/LEF, transcription factors involved in the Wnt signaling pathway.

Elderly patients with advanced CKD have to face with the difficult decision-making about dialysis initiation as renal function declines toward ESRD. The choices have become even tougher in the wake of escalating financial burden imposed by the long-term nature of treatment. Most guidelines advocate to postpone dialysis initiation until the eGFR has reached 5-10 or ≤6 ml/min/1.73 m^2^ for asymptomatic patients, i.e., deferring the initiation of long-term dialysis until absolutely indicated [125]. This paradigm shift is supported by the IDEAL study [126], and a subsequent study exploring the optimal timing of dialysis initiation among older adults with advanced CKD which showed that compared with medical management, dialysis initiation at eGFR <6 ml/min/1.73 m^2^associated with a higher median life expectancy of 26, 25, and 19 months for patients aged 65, 75, and 85 years, respectively [127]. Conversely, conservative kidney management without provision of dialysis may be an alternative option for some older patients with poor health conditions. For example, in a study included 349,440 patients aged 70 years and over at the time of initiation of dialysis, institutionalized individuals had higher mortality as compared to non-instutionalized counterparts in the same age group. The researchers concluded that for frail elderly with advanced CKD, especially those with multiple comorbidities, or major neurocognitive disorders, conservative management with palliative or end-of-life care should be considered due to lack of therapeutic benefits of dialysis therapy [128]. Other studies have also observed that patients in nursing home or higher severity of frailty at dialysis initiation are associated with a substantial, post-dialysis decline in functional status and patient survival, respectively [129, 130]. Thus, with backup from palliative care professionals, later start of dialysis or use of conservative management, including reduced dose or frequency of dialysis, may be considered for patients complicated by severe functional or cognitive impairments [45, 130, 131]. In contrast, for robust elderly patients with ESRD whose activities of daily living remain independent, there is no reason why long-term dialysis therapy should not be initiated according to standard treatment guidelines (Fig. 1).

## Conclusions and perspectives

The aged kidney undergoes structural and functional changes which are indistinguishable from that of incipient kidney diseases. Differentiating these two conditions is crucial in implementing available strategies to prevent the development of kidney disease. Apart from mechanistic research need on kidney senescence, there exist knowledge gaps in caring for older patients with CKD. First, most clinical trials for renal progression have excluded older patients, leading to competing therapeutic priorities, and the resultant clinical guidelines might not be generalizable to all persons involved. Second, elderly people are vulnerable to superimposed AKI, yet effective measures or treatment tackling AKI to CKD transition or AKI on CKD progression have not been explored to the fullest extent possible. Last but not least, it remains a challenge for clinicians when it comes to the decision-making of dialysis initiation. Studies addressing the risk of mortality, quality of life and functional capacity in geriatric patients are desperately required so that those who are robust can opt for dialysis whereas those complicated by existing functional or cognitive impairments may consider conservative management.
